# Supplementary material for: The Pandemic-Related Factors Associated with Emergency Department Visits in Portugal throughout Two Years of the Pandemic: A Retrospective Population-Based Study
Source: Int J Environ Res Public Health. 2023 Jan 10;20(2):1207. doi: 10.3390/ijerph20021207 (PMC9858921; doi:10.3390/ijerph20021207)

Supplementary material

Contents

Figure S1: ED admissions in mainland Portugal 3
Figure S2: ED admissions in the North 4
Figure S3: ED admissions in LVT 5
Figure S4: ED admissions in the centre 6
Figure S5: ED admissions in Algarve 7
Figure S6. ED admission in Alentejo 8
Figure S7: Incidence rate of COVID-19 per 10k inhabitants in mainland Portugal 9
Figure S8: Incidence rate of COVID-19 per 10k inhabitants in the North 10
Figure S9: Incidence rate of COVID-19 per 10k inhabitants in the Centre 11
Figure S10: Incidence rate of COVID-19 per 10k inhabitants in LVT 12
Figure S11: Incidence rate of COVID-19 per 10k inhabitants in Algarve 13
Figure S12: Incidence rate of COVID-19 per 10k inhabitants in Alentejo 14
Figure S13: Vaccination coverage in mainland Portugal 15
Figure S14: Vaccination coverage in the North 16
Figure S15: Vaccinatin coverage in the Centre 17
Figure S16: Vaccination coverage in LVT 18
Figure S17: Vaccination coverage in Algarve 19
Figure S18: Vaccination coverage in Alentejo 20
Figure S19: Containment index in Portugal 21
Figure S20: Google search on the term coronavirus in Portugal 22
Figure S21: Mobility in Portugal 23

Figure S1: ED admissions in mainland Portugal

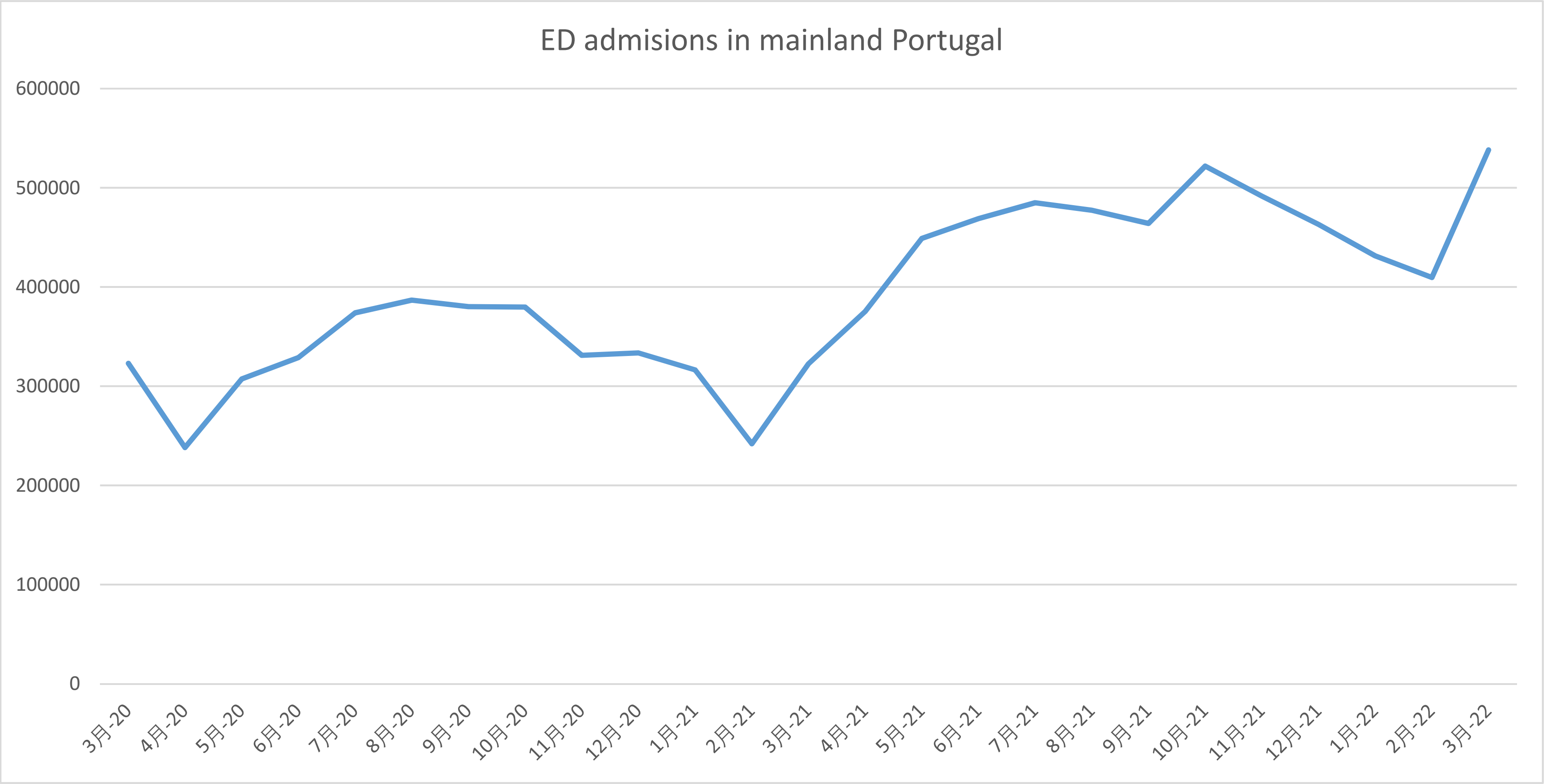

Figure S2: ED admissions in the North

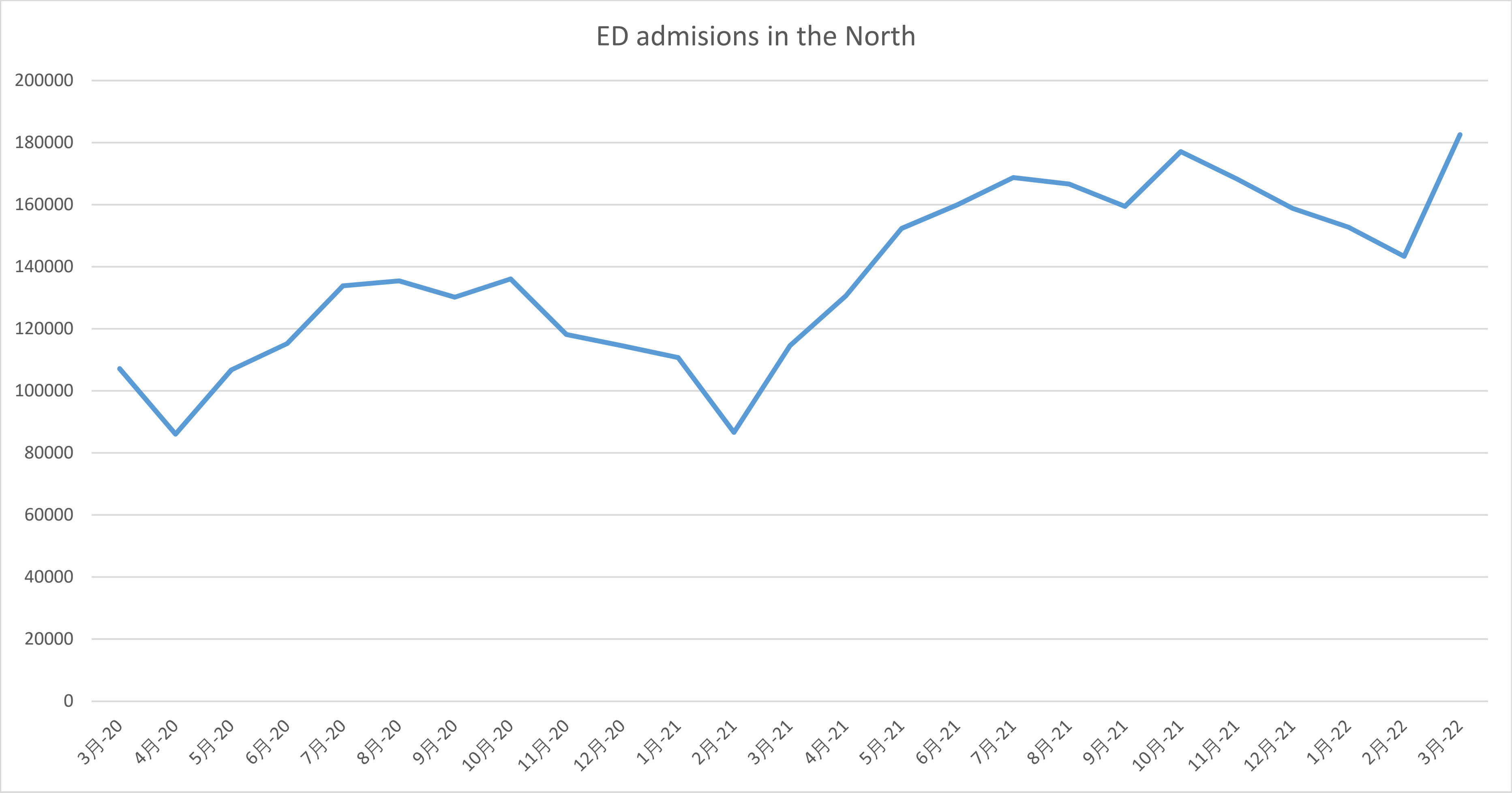

Figure S3: ED admissions in LVT

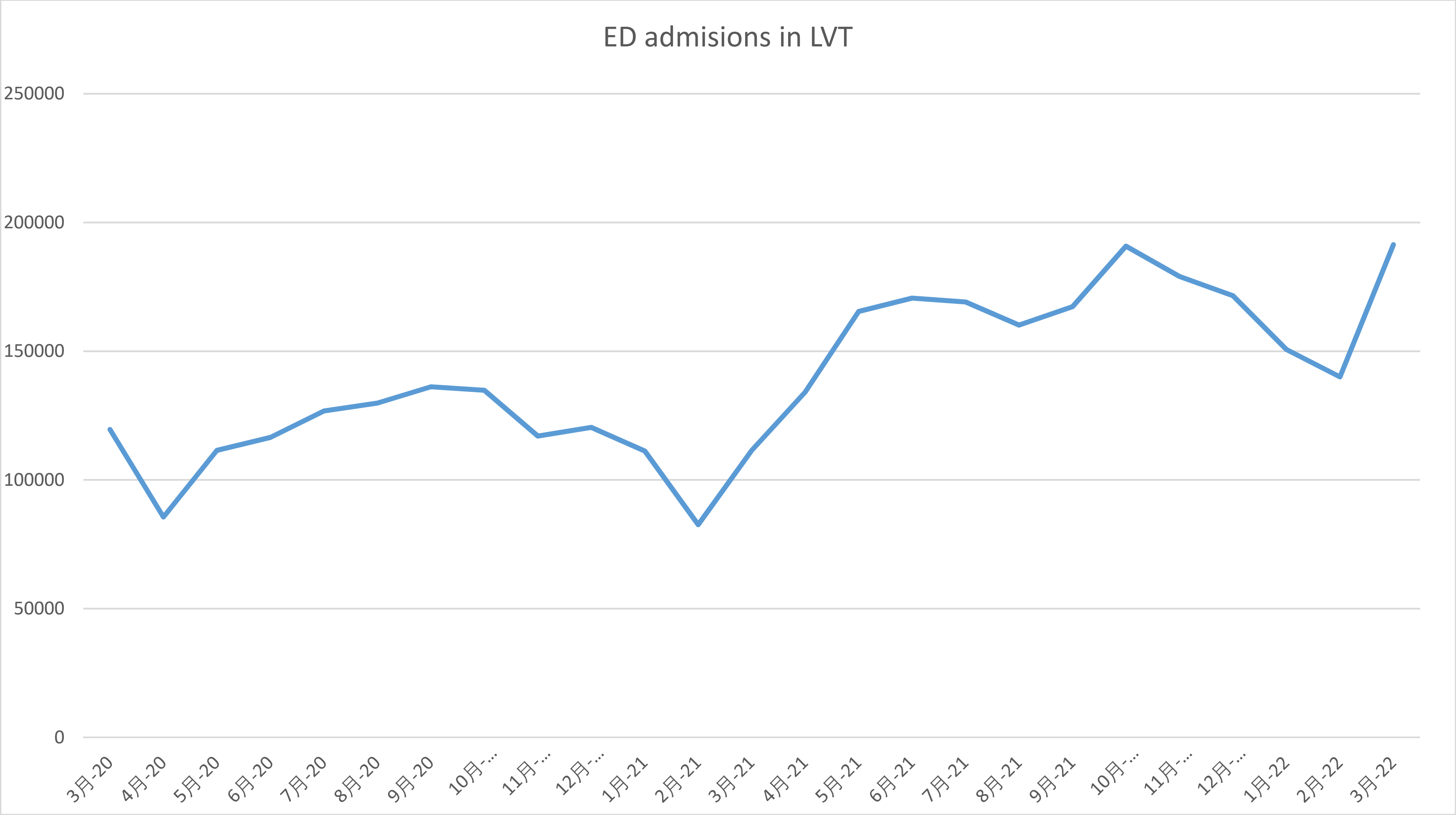

Figure S4: ED admissions in the centre

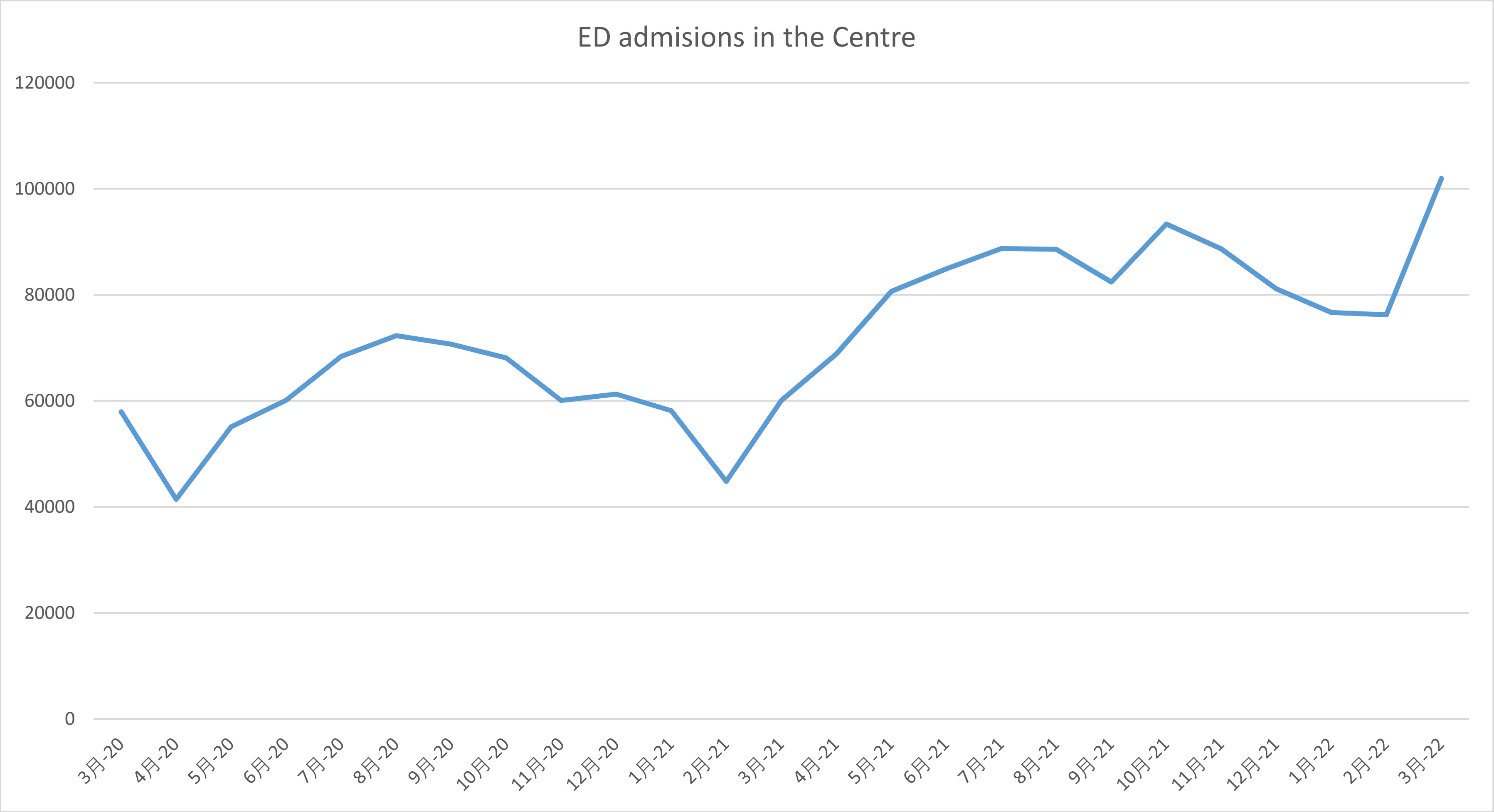

Figure S5: ED admissions in Algarve

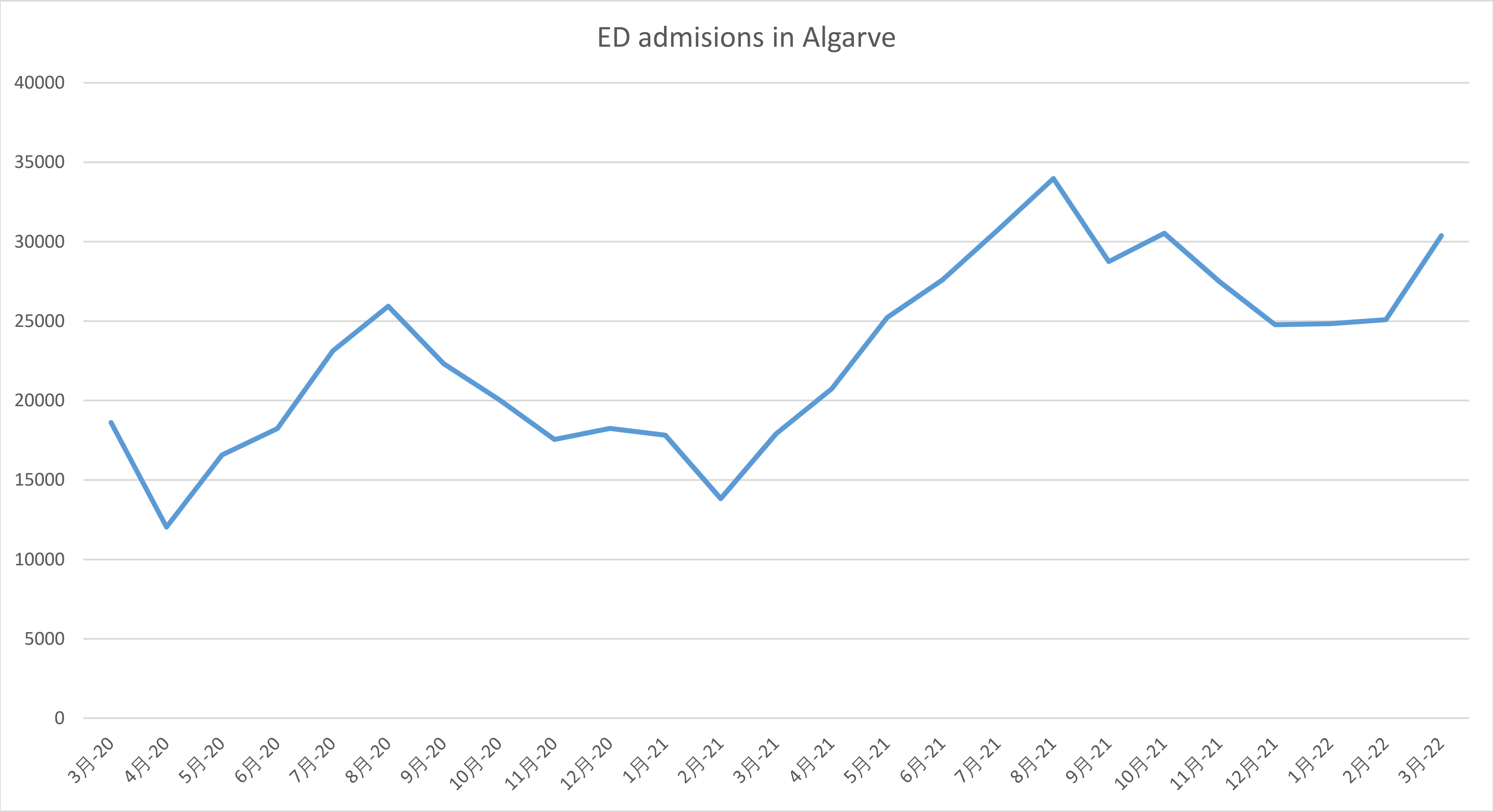

Figure S6. ED admission in Alentejo

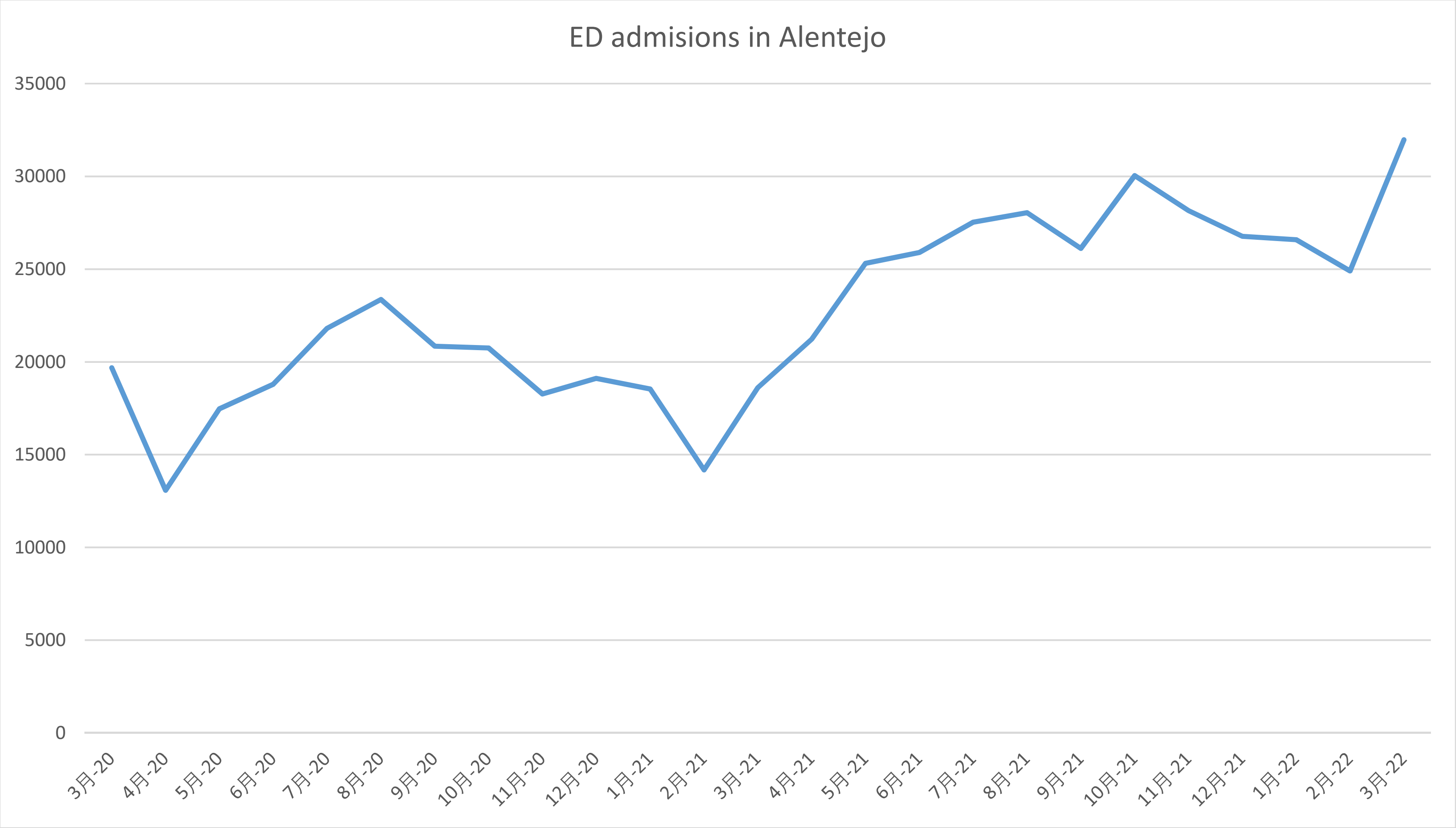

Figure S7: Incidence rate of COVID-19 per 10k inhabitants in mainland Portugal

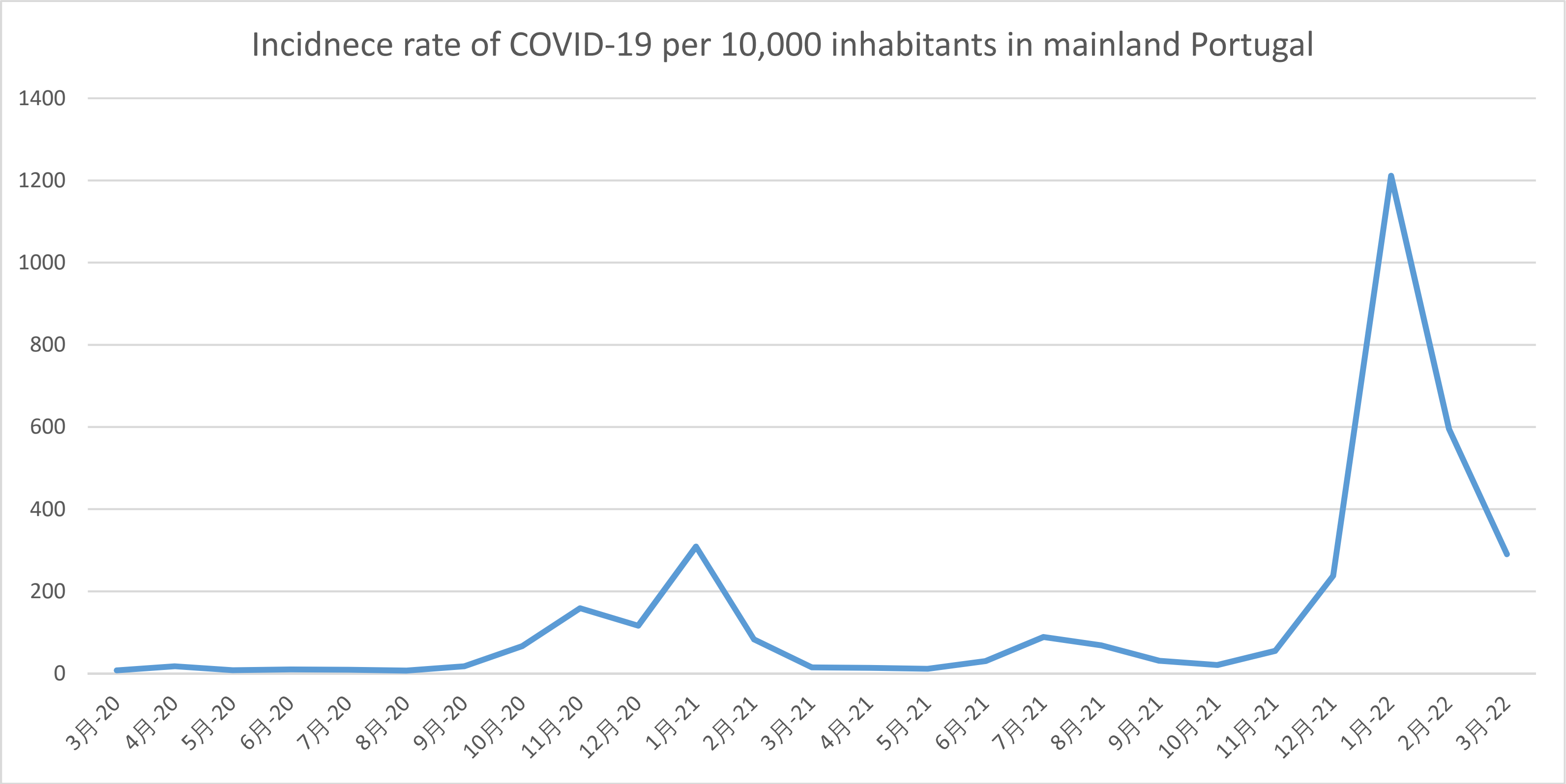

Figure S8: Incidence rate of COVID-19 per 10k inhabitants in the North

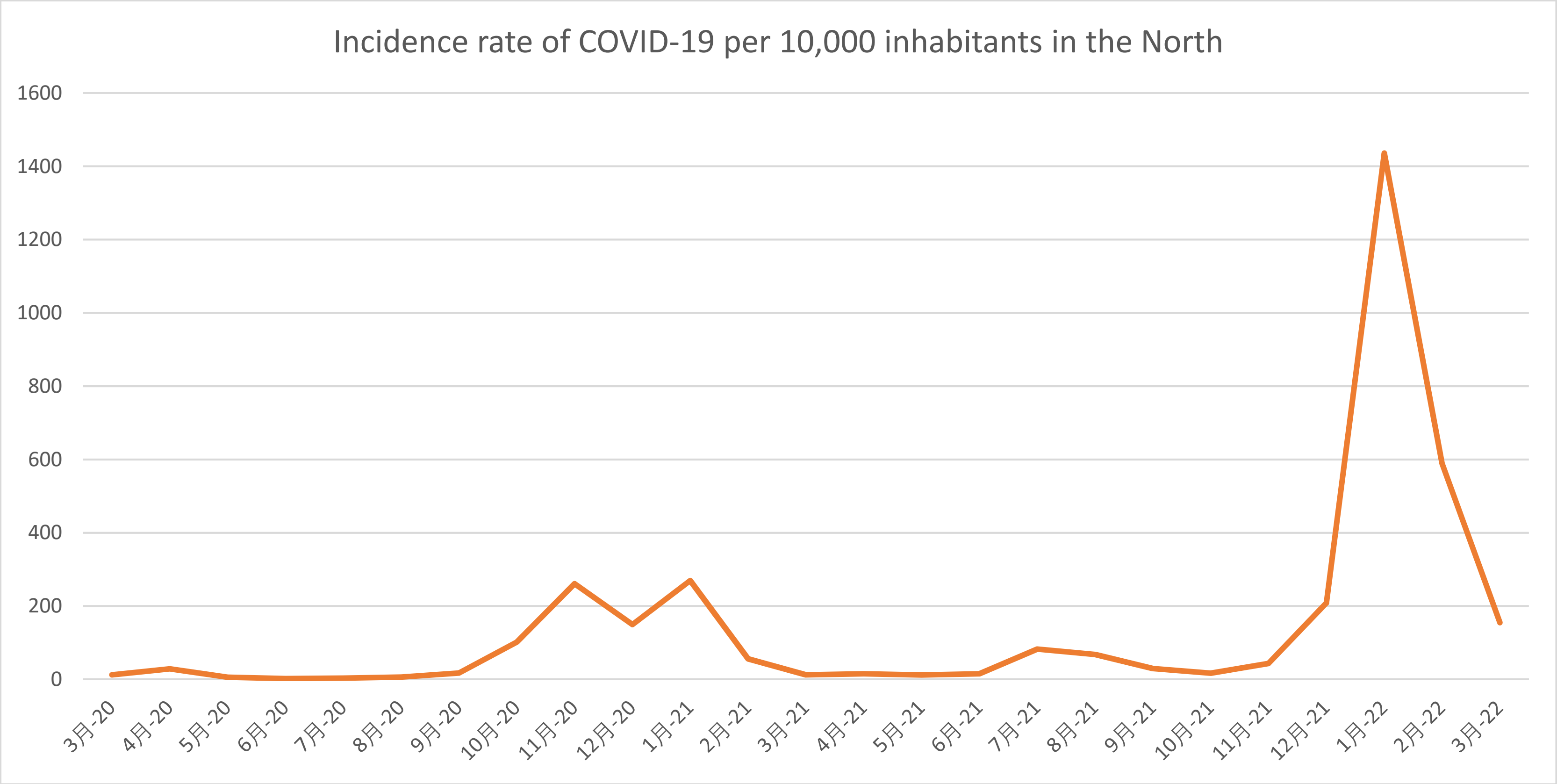

Figure S9: Incidence rate of COVID-19 per 10k inhabitants in the Centre

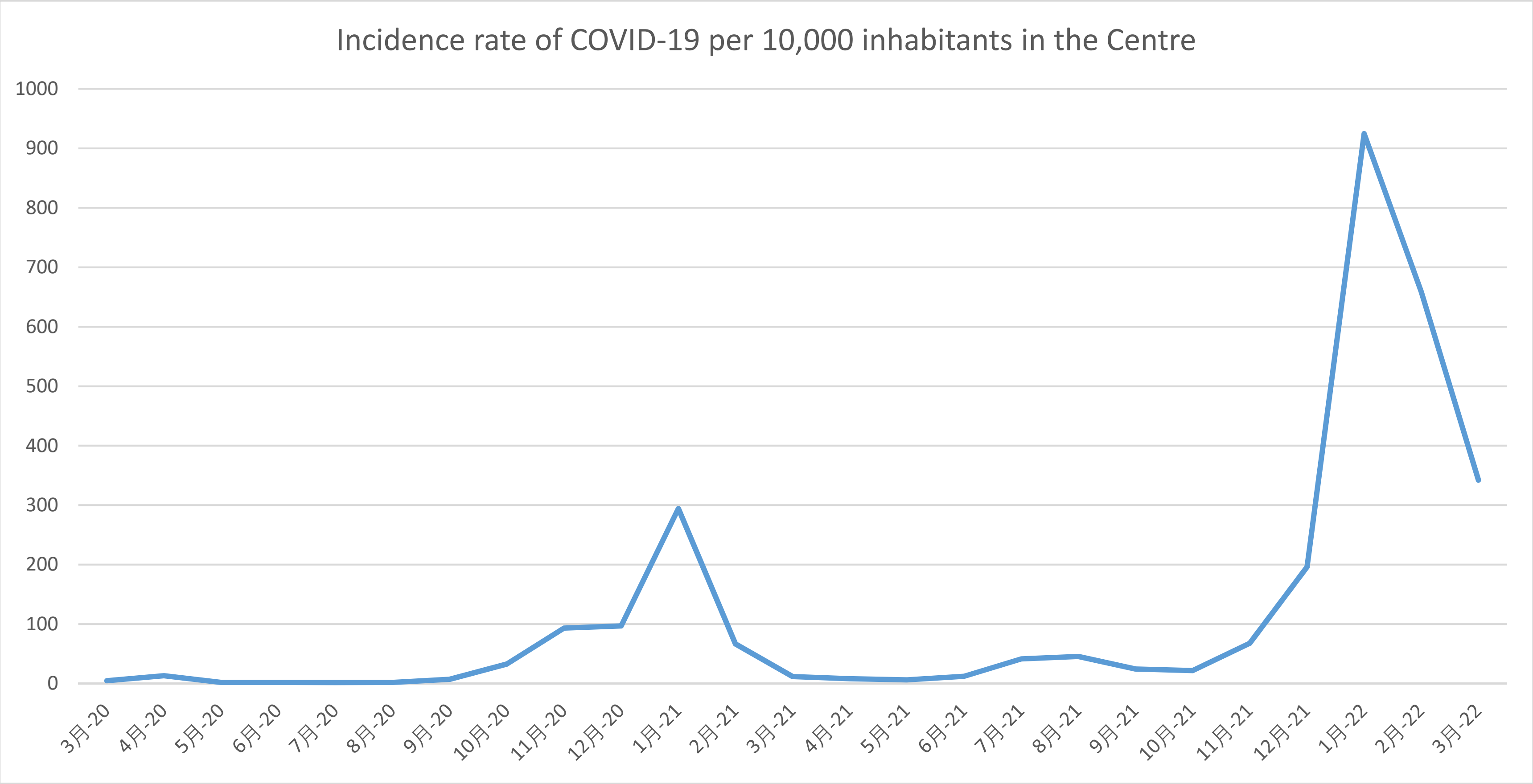

Figure S10: Incidence rate of COVID-19 per 10k inhabitants in LVT

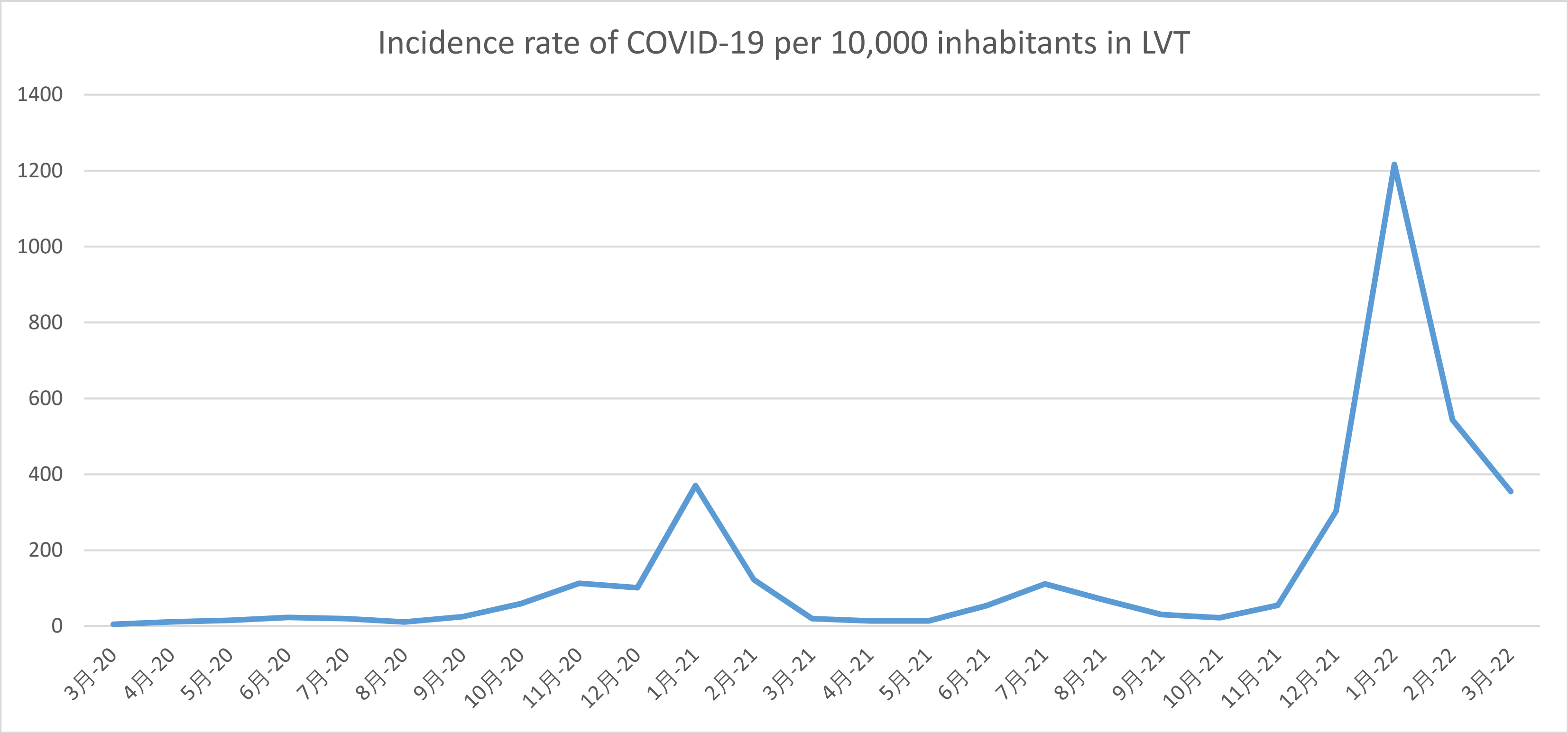

Figure S11: Incidence rate of COVID-19 per 10k inhabitants in Algarve

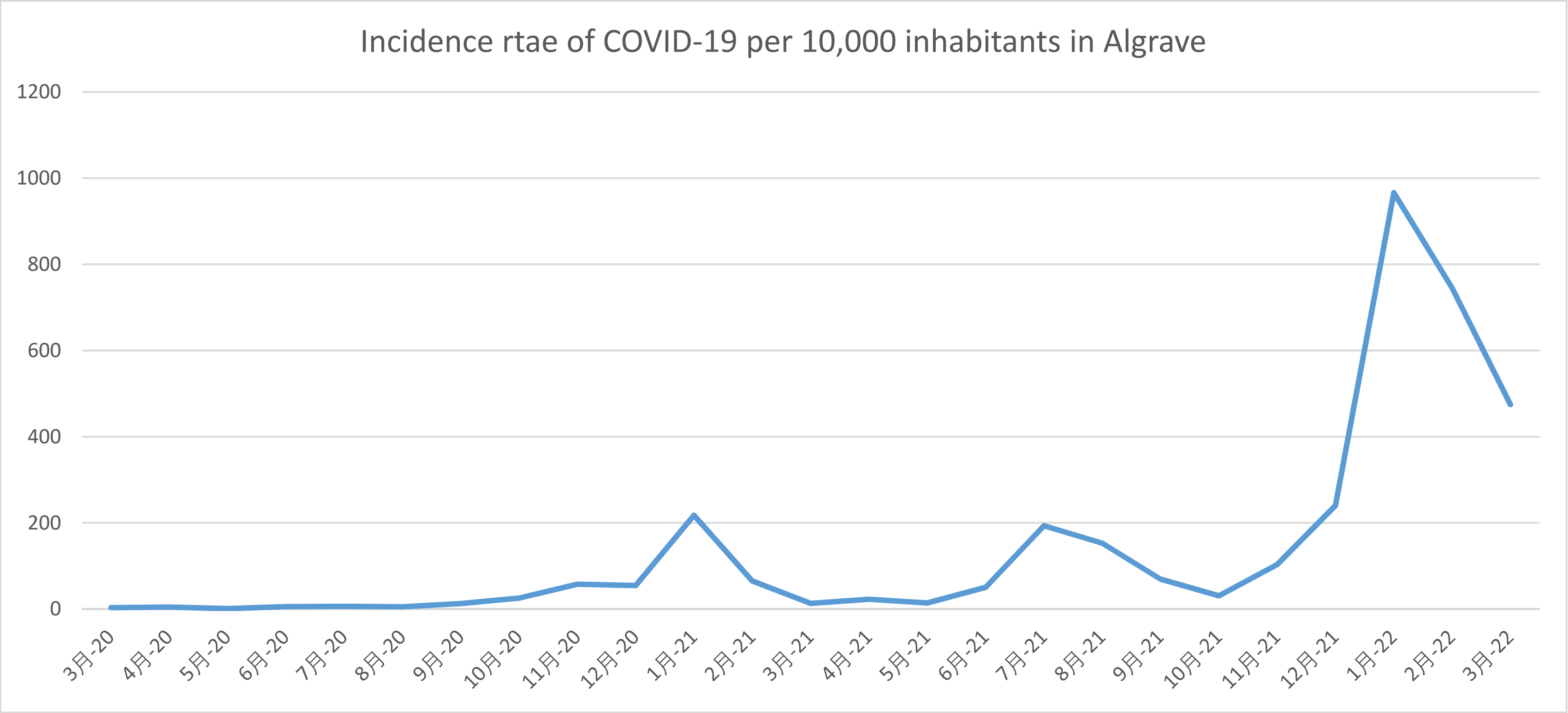

Figure S12: Incidence rate of COVID-19 per 10k inhabitants in Alentejo

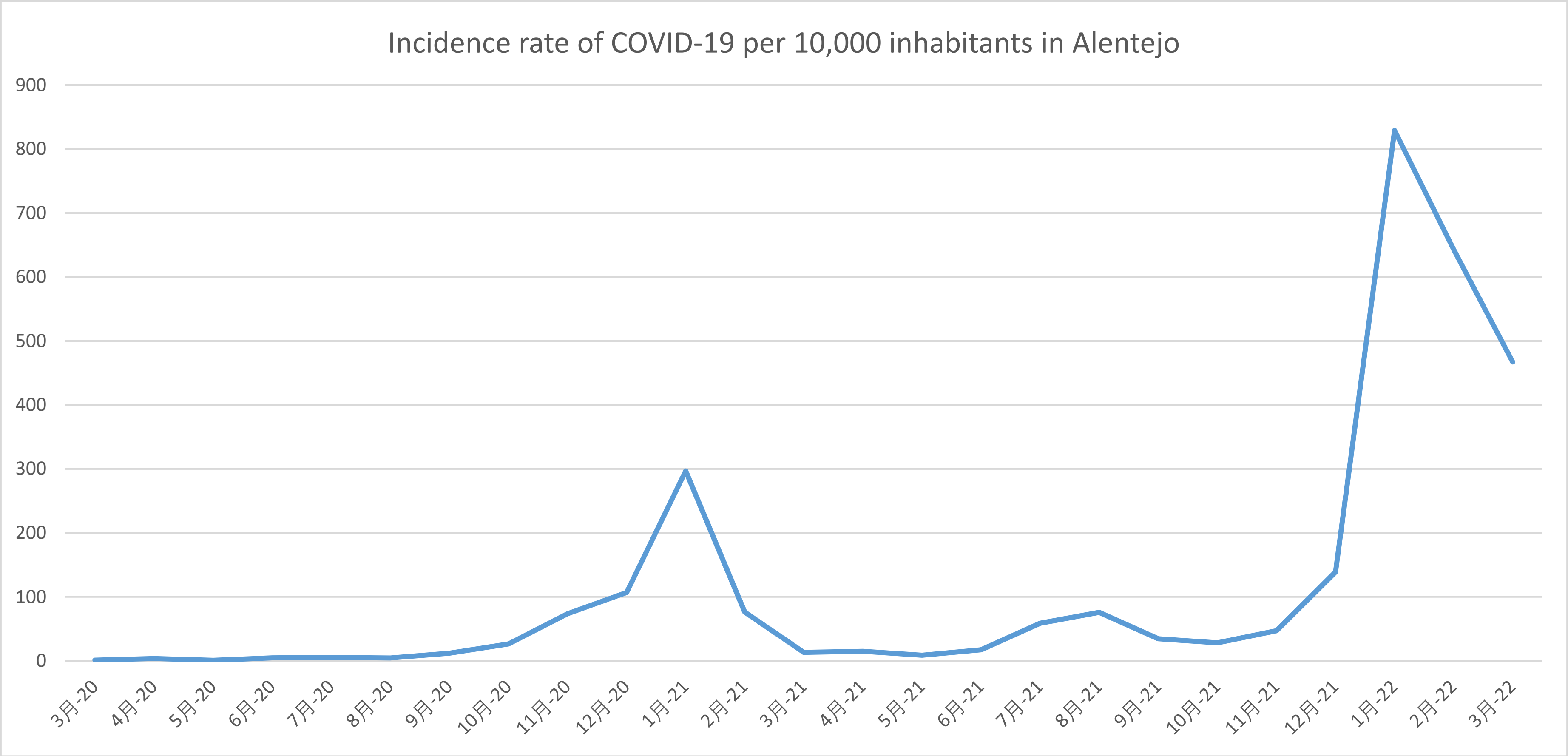

Figure S13: Vaccination coverage in mainland Portugal

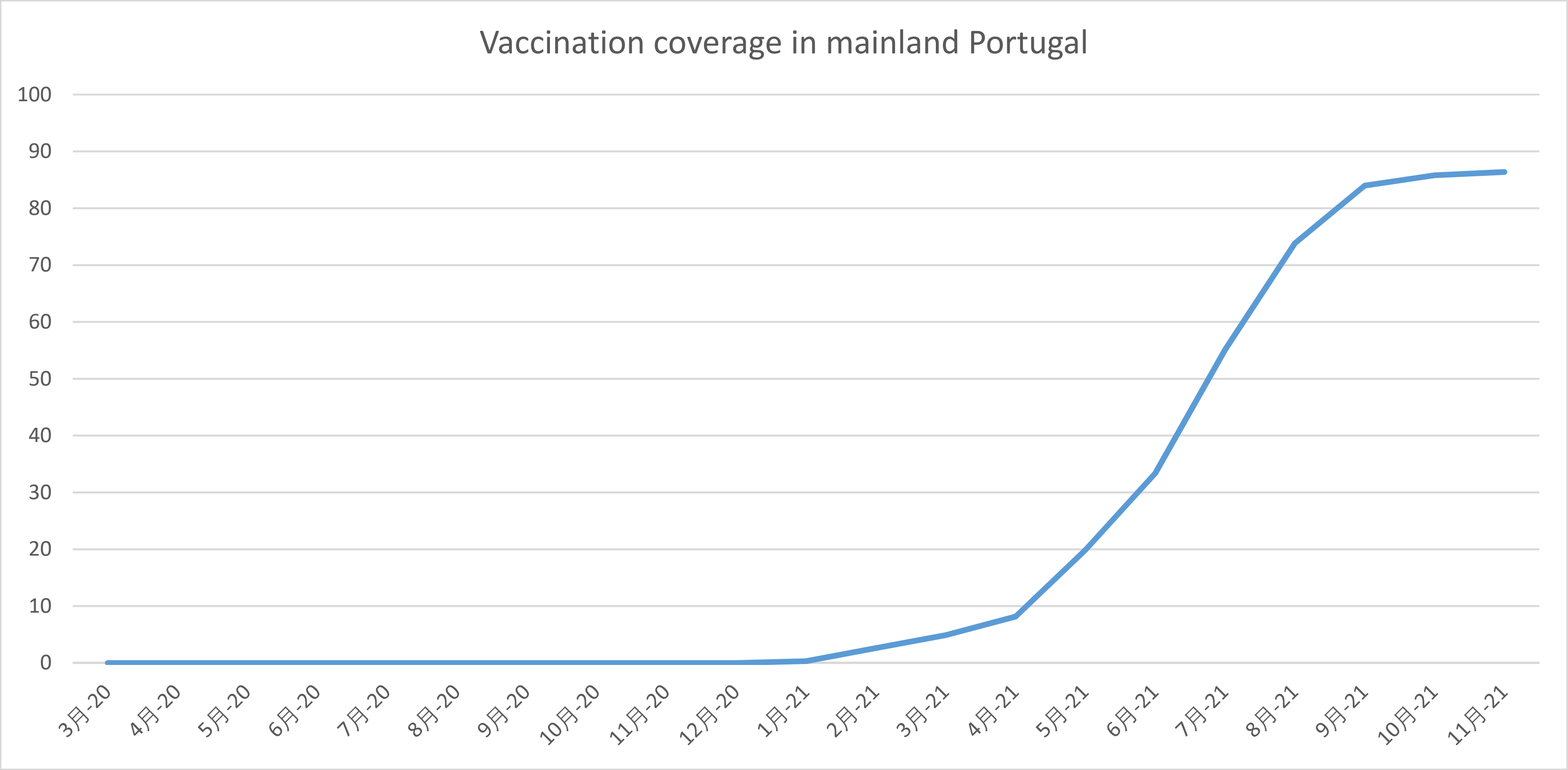

Figure S14: Vaccination coverage in the North

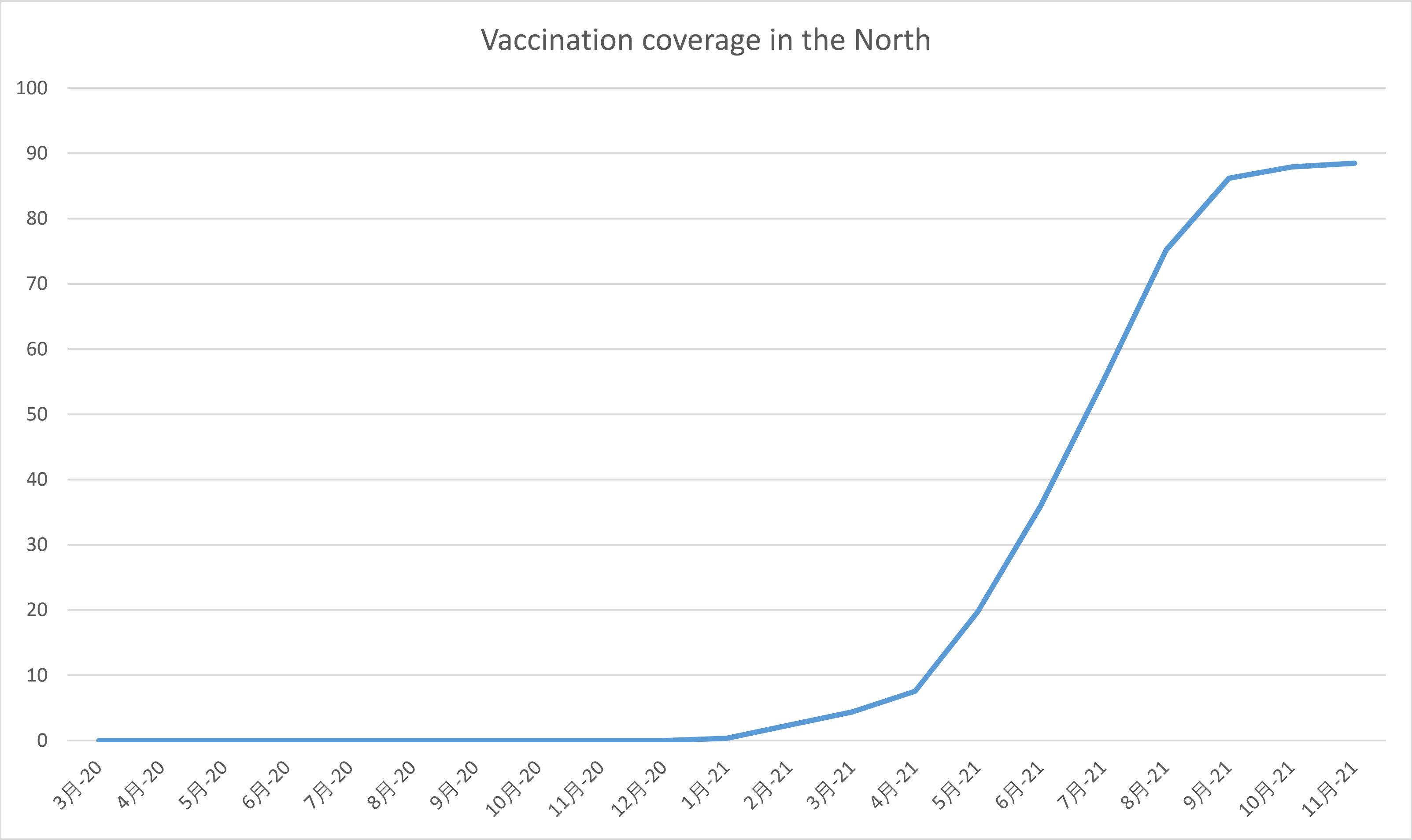

Figure S15: Vaccinatin coverage in the Centre

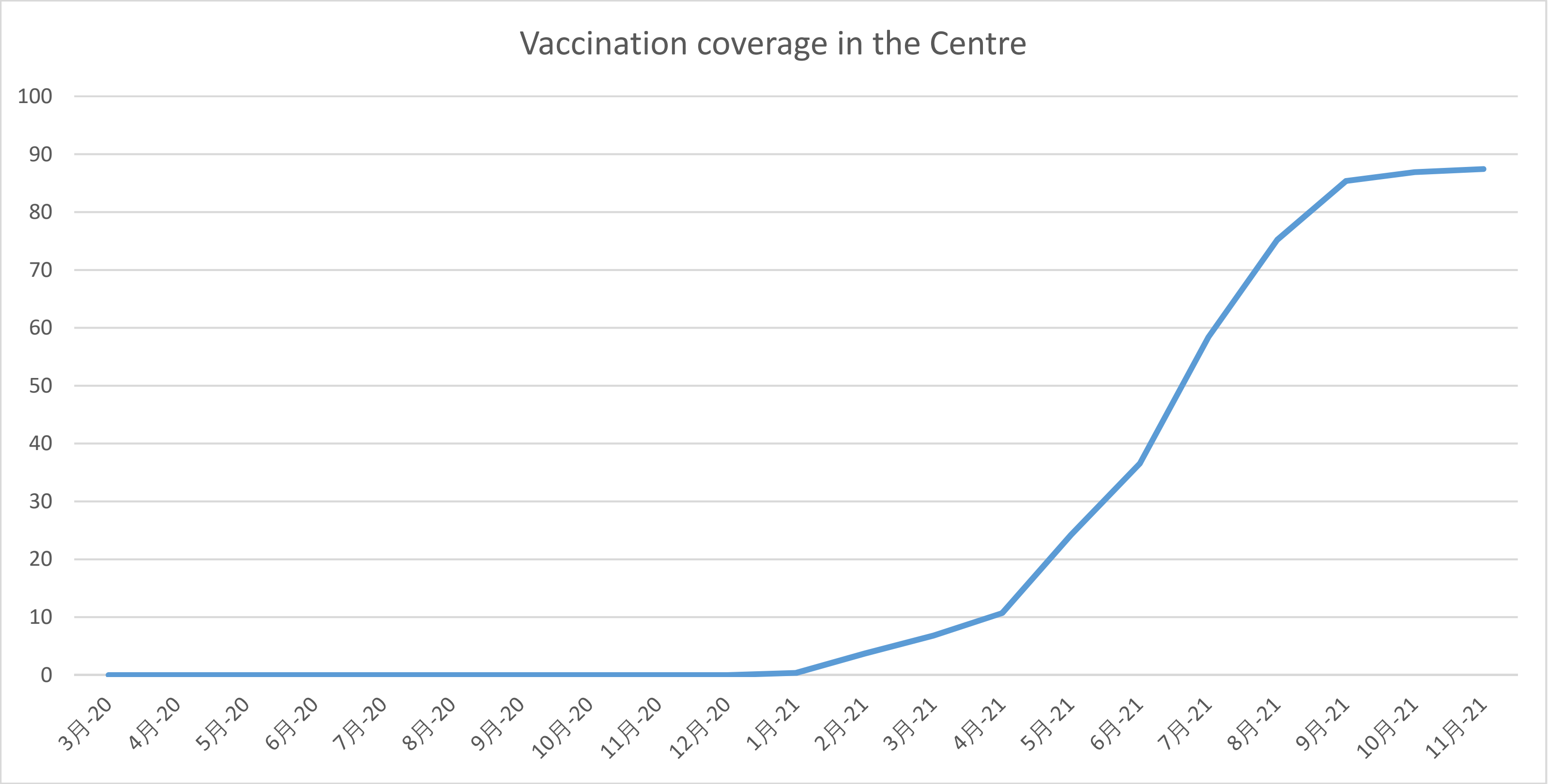

Figure S16: Vaccination coverage in LVT

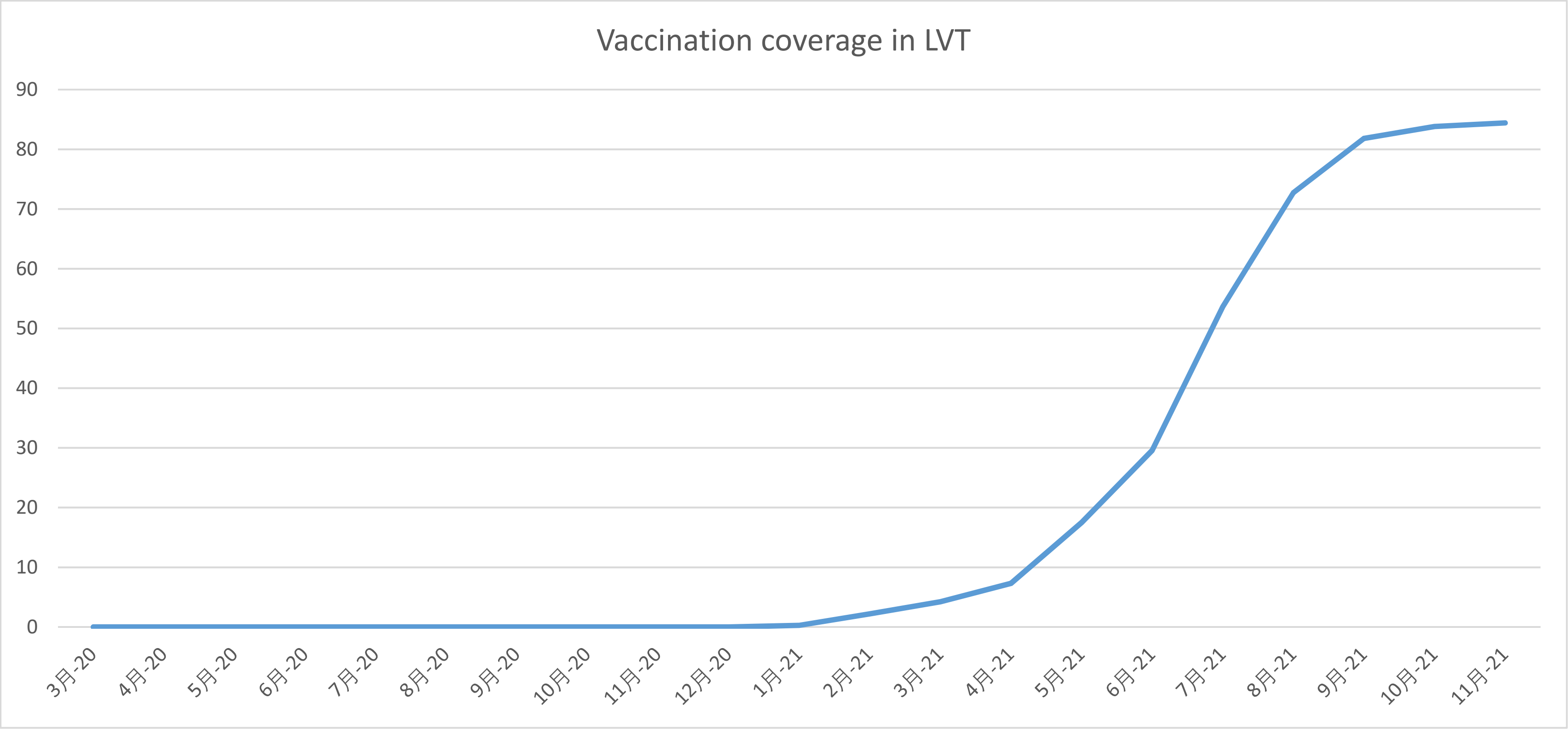

Figure S17: Vaccination coverage in Algarve

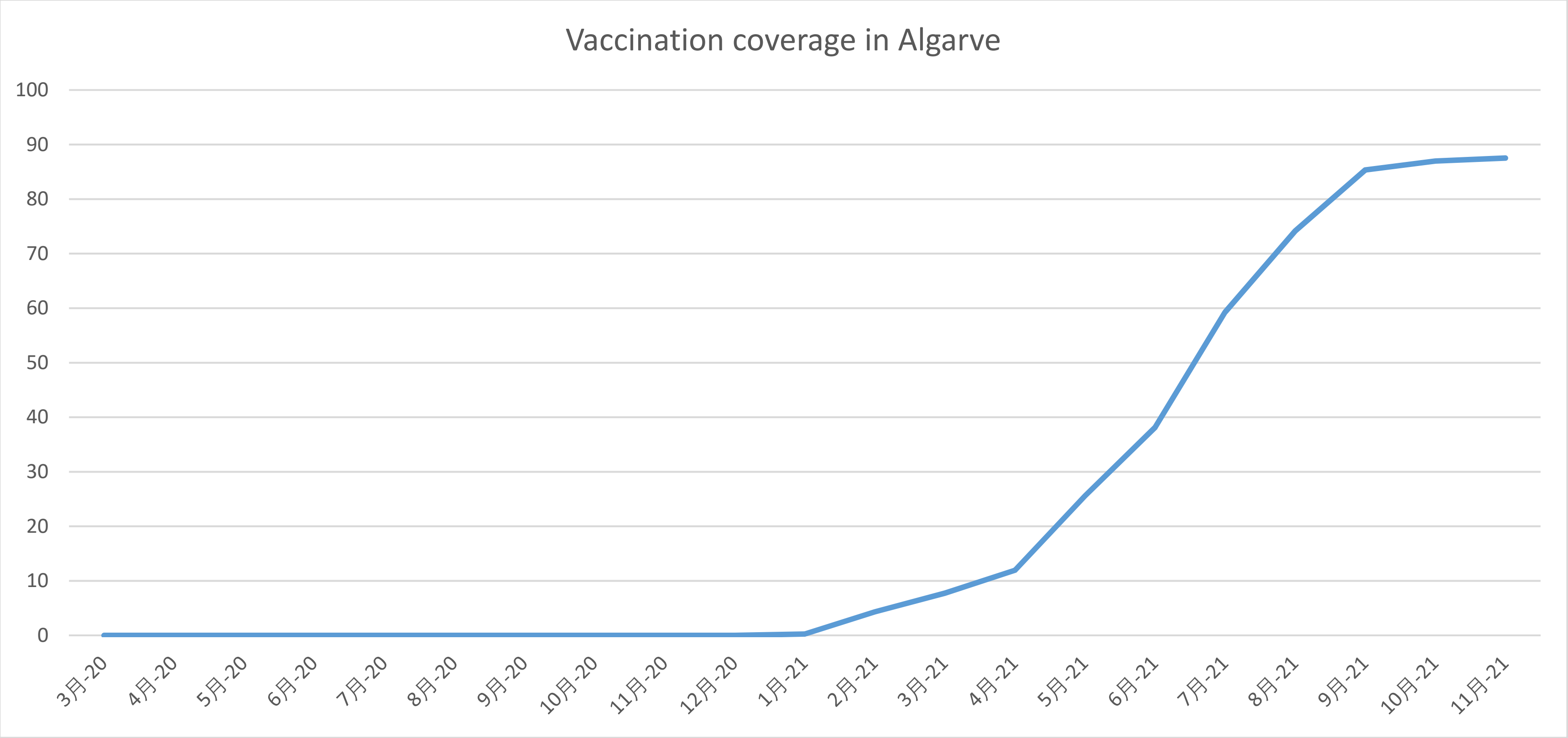

Figure S18: Vaccination coverage in Alentejo

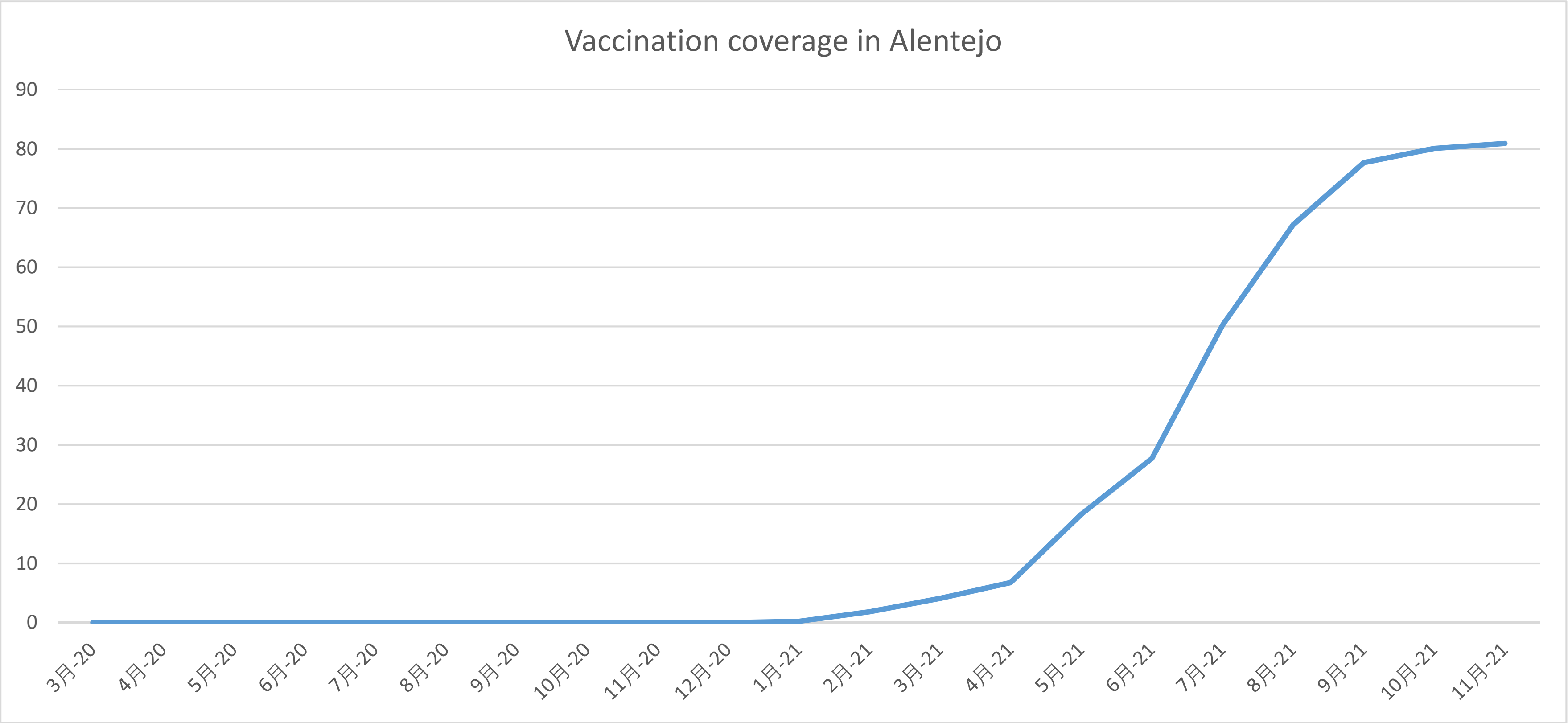

Figure S19: Containment index in Portugal

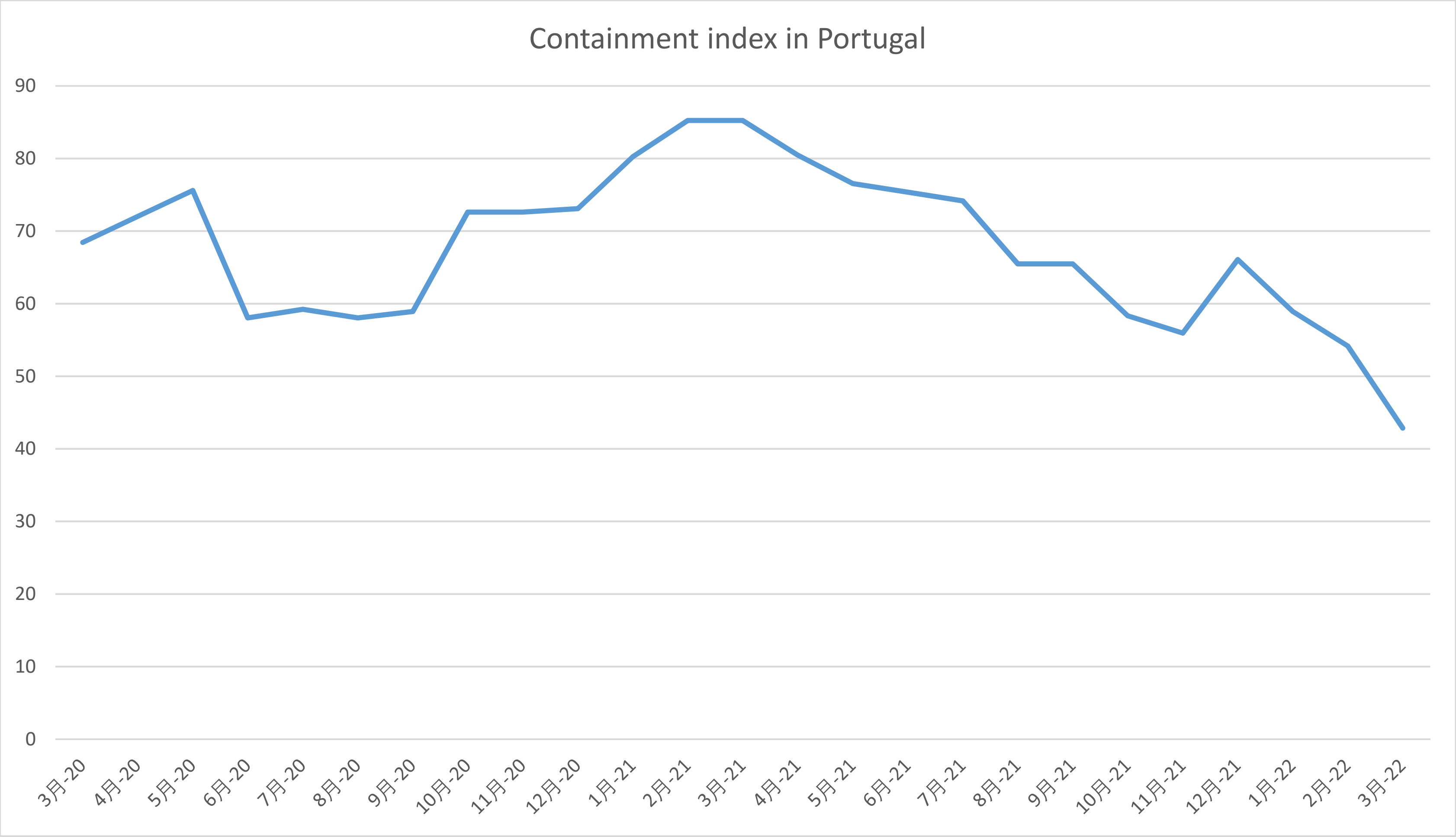

Figure S20: Google search on the term coronavirus in Portugal

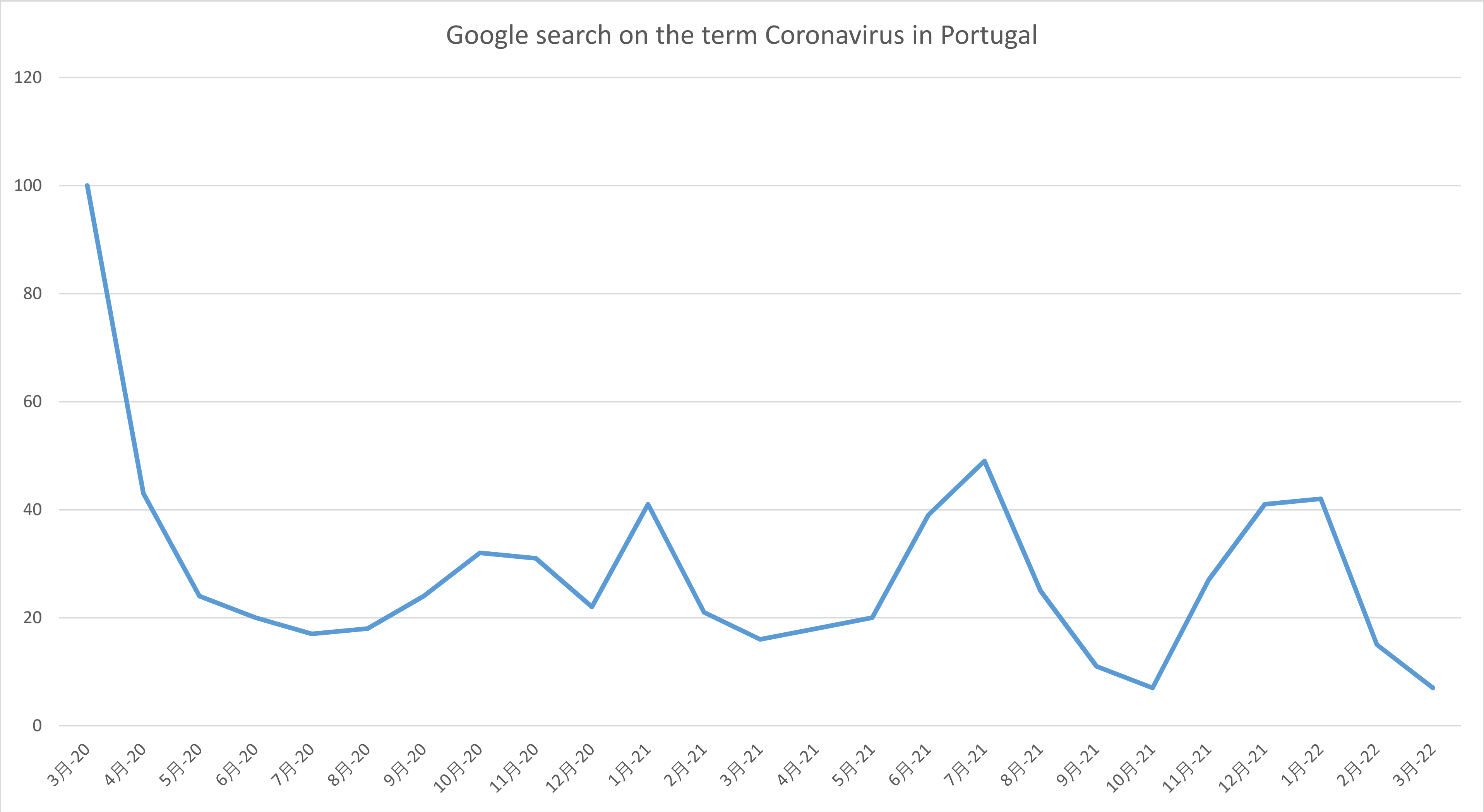

Figure S21: Mobility in Portugal

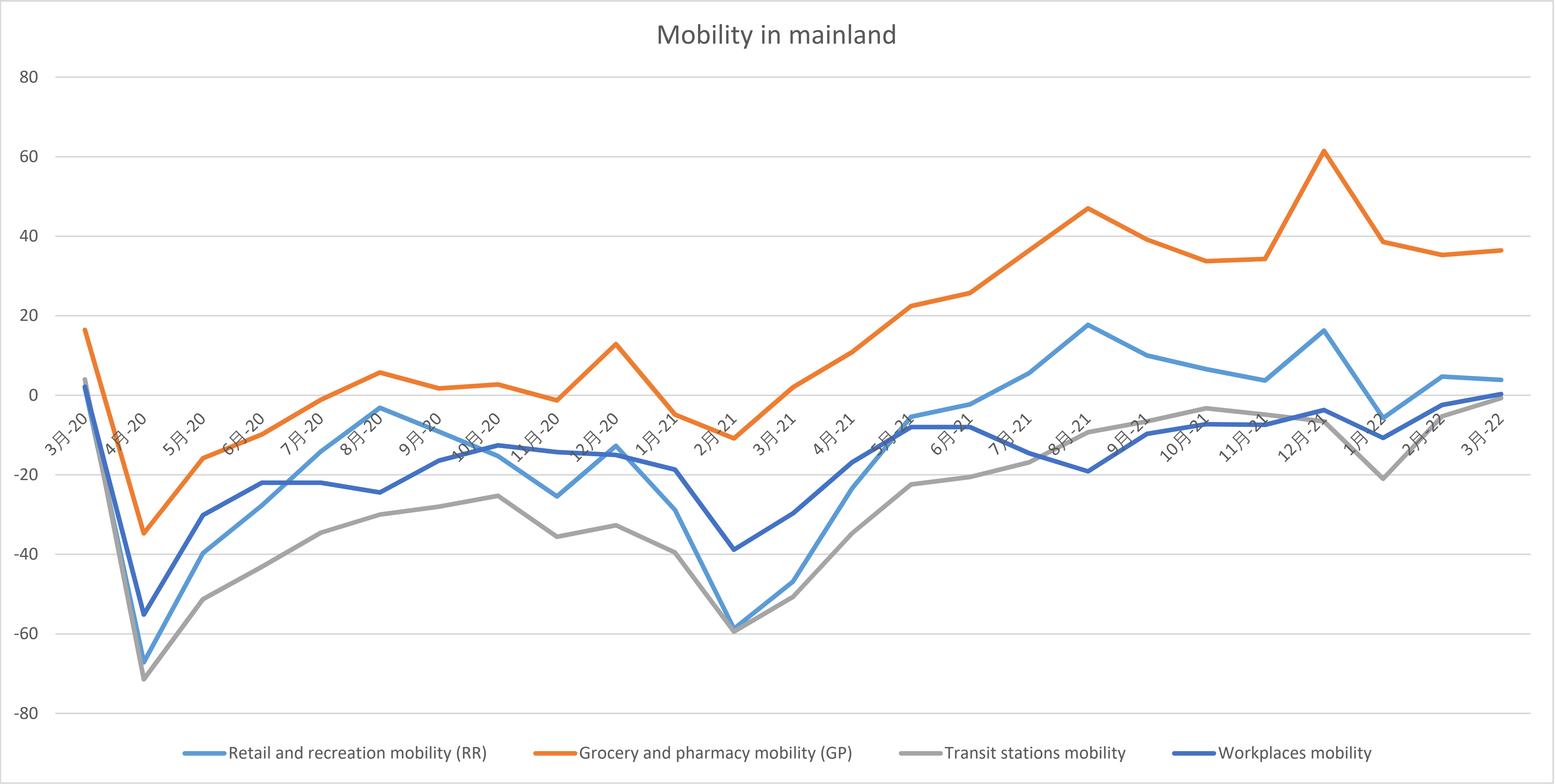

Supplement: Supplementary file 1 [file ijerph-20-01207-s001.zip › ijerph-2049964-supplementary.pdf]
